# A qualitative study regarding older people’s goals of care in relation to frailty status: finding meaning in ‘smaller things’ in life

**DOI:** 10.1093/ageing/afaf022

**Published:** 2025-02-20

**Authors:** Veerle M G T H van der Klei, Frederiek van den Bos, Simon P Mooijaart, Anneke G Julien, Mabel J E Maissan, Bas F M van Raaij, Jan Festen, Jacobijn Gussekloo, Yvonne M Drewes

**Affiliations:** Department of Internal Medicine, Section of Gerontology and Geriatrics, Leiden University Medical Center, Leiden, Netherlands; LUMC Center for Medicine for Older People (LCO), Leiden University Medical Center, Leiden, Netherlands; Department of Internal Medicine, Section of Gerontology and Geriatrics, Leiden University Medical Center, Leiden, Netherlands; LUMC Center for Medicine for Older People (LCO), Leiden University Medical Center, Leiden, Netherlands; Department of Internal Medicine, Section of Gerontology and Geriatrics, Leiden University Medical Center, Leiden, Netherlands; LUMC Center for Medicine for Older People (LCO), Leiden University Medical Center, Leiden, Netherlands; Department of Internal Medicine, Section of Gerontology and Geriatrics, Leiden University Medical Center, Leiden, Netherlands; LUMC Center for Medicine for Older People (LCO), Leiden University Medical Center, Leiden, Netherlands; Department of Internal Medicine, Section of Gerontology and Geriatrics, Leiden University Medical Center, Leiden, Netherlands; LUMC Center for Medicine for Older People (LCO), Leiden University Medical Center, Leiden, Netherlands; Department of Internal Medicine, Section of Gerontology and Geriatrics, Leiden University Medical Center, Leiden, Netherlands; LUMC Center for Medicine for Older People (LCO), Leiden University Medical Center, Leiden, Netherlands; KBO, Bemmel Gelderland, Netherlands; Department of Internal Medicine, Section of Gerontology and Geriatrics, Leiden University Medical Center, Leiden, Netherlands; LUMC Center for Medicine for Older People (LCO), Leiden University Medical Center, Leiden, Netherlands; Department of Public Health and Primary Care, Leiden University Medical Center, Leiden, Netherlands; Department of Internal Medicine, Section of Gerontology and Geriatrics, Leiden University Medical Center, Leiden, Netherlands; LUMC Center for Medicine for Older People (LCO), Leiden University Medical Center, Leiden, Netherlands

**Keywords:** older people, decision-making, patient preference, quality of life, patient and public involvement, qualitative research

## Abstract

**Background:**

Increasingly frailty assessment is part of the shared decision-making process of older patients. However, little is known of the role of frailty in goals of care among the diverse group of older persons.

**Objective:**

To explore the role of frailty in older people’s perspectives on goals of care in case of acute and/or severe disease.

**Methods:**

We conducted semi-structured interviews with people aged ≥70 years in the Netherlands (*n* = 26), which were purposively sampled based on a self-reported Clinical Frailty Scale. The interviews were analysed using thematic content analysis to compare frailty subgroups.

**Results:**

Three themes regarding goals of care emerged: (1) preserving well-being in one’s lifeworld through life goals; (2) goals related to care, as access to appropriate care, good contact with care professionals and a dignified end-of-life; (3) differences in attainment and adaptation of goals of care according to frailty status. The first two themes appeared to be independent of frailty status. However, differences were seen in theme 3, as fit older people primarily strengthened their capacity to attain goals of care, while frail older people primarily adapted the meaning ascribed to goals of care and had higher acceptance of the life cycle.

**Conclusion:**

Goals of care that older people want to attain are driven by life goals, independent of frailty. Therefore, older people with varying frailty status could be treated similarly in goal-setting and life goals. However, different support may be needed for the attainment and adaptation of their goals of care.

## Key Points

- Fit and frail older people’s types of goals of care are similar, where their prioritised life goals are more personal.
- Continuing current life when being ill by maintaining these life goals was essential for all to attain person-centred care.
- Older people with varying frailty status could be treated similarly in goal-setting by eliciting personal meaning of goals.
- The way fit and frail older people attained and adapted their goals of care differed (e.g. by increasingly ‘smaller things’).
- Different attention of a wide range of care professionals might be needed to support their attainment or adaptation strategy.

## Introduction

Person-centredness is a cornerstone of high-quality health care in the twenty-first century [1]. While the preceding disease-oriented health care systems primarily focused on ‘What is the matter with you?’, person-centred care in addition focuses on ‘What matters to you?’ [2, 3]. Goals of care, defined as the overarching aims of medical care for a patient [4], therefore ideally incorporate the perspectives of patients themselves about what matters to them when being ill [3, 5, 6]. Especially for shared decision-making (SDM), personal goals of care are important to take into account. Unfortunately, discordance in goal-setting tends to occur between the perspective of patients and the perspective of their doctors [6, 7], which emphasises the need for exploring the underlying drivers of patients’ goals of care in research.

Eliciting patient’s goals of care in SDM and aligning health care accordingly is especially important when treating older patients because of the large heterogeneity in older people’s health and frailty status [8]. As frailty is known to be associated with adverse health outcomes (e.g. decline in functioning, quality of life and mortality) [9], doctors across the health care system increasingly integrate frailty assessment as an important aspect of the decision-making process in older patients [10]. Besides frailty assessment, personalised treatment goals are another important aspect in SDM with older patients. However, little is known about how older people’s goals of care relate to their frailty status, which matters in case of acute and/or severe disease, as was illustrated during the COVID-19 pandemic, when patients appeared to be at risk to receive care that was not completely aligned with their actual wishes [11]. This lack of relevant scientific evidence regarding older patients leads to uncertainty for doctors in making personalised treatment recommendations [8, 12].

As research team, we hypothesised that older people’s goals of care were likely to differ with increasing frailty status (e.g. transitioning from a focus on life extension towards quality of life and comfort-focused care), driven by disease experiences [7]. However, our recent quantitative study among the general older population (70+) surprisingly showed that older people’s preferred goals of care in case of acute and/or severe disease were not related to frailty status [13]. In short, the most important goals of older people were preventing nursing home admission, staying independent and preserving quality of life, while extending life was relatively unimportant, which was similar across frailty subgroups [13]. These findings were in line with the limited available evidence from questionnaire-based clinical studies in specific subsets of older people such as hip fracture patients and patients with head and neck cancer [14–16] as these studies also showed no difference in goals of care between non-frail and frail geriatric patients. To further explain these quantitative findings and to investigate the underlying drivers, as well as the possible mismatch between the perspectives of professionals and older people themselves [17], we aimed to further explore the role of frailty in older people’s goals of care in case of acute and/or severe disease.

## Methods

### Study design and participants

This qualitative interview study is part of a mixed-methods study on older people’s goals of care, which is embedded in the *COVID-19 Outcomes in Older People* (COOP) consortium. The study population of our preceding quantitative survey study (*n* = 1278) [13], in which anyone aged ≥70 years in the Netherlands could participate, was used as a sampling frame. In this survey, frailty was assessed by 11 self-reported, closed-ended questions to derive a self-reported Clinical Frailty Scale (CFS) [13]. Out of those who were willing to be interviewed (*n* = 496), older people were purposively sampled based on data from the quantitative questionnaire. We invited a heterogenous sample regarding frailty status: from fit (self-reported CFS 1–3) to mildly frail (self-reported CFS 4–5) and severely frail (self-reported CFS 6–8). We additionally aimed to reach a maximum variation sample on sociodemographics and experienced health problems. The study was approved by the Institutional Review Board of the Leiden University Medical Center for observational COVID-19 studies (2022-005), and all participants provided verbal informed consent. More details on the larger COOP consortium have been described previously [13]. The consolidated criteria for reporting qualitative research (COREQ) checklist was used to guide this article [18].

### Data collection

Four members of the research team conducted the interviews, who had a professional background in medicine (V.v.d.K., M.M., B.v.R.), sociology (A.J.), and vitality and ageing (V.v.d.K. and A.J.). All were formally trained in qualitative interviewing. The research team additionally consisted of senior researchers with diverse backgrounds in medicine for older people [i.e. community medicine (Y.D.), general practitioner (J.G.) and internist-geriatricians (F.v.d.B. and S.M.)]. The team as a whole had extensive experience in qualitative research, and no one was simultaneously involved in the medical care of the interviewees.

Older people were approached by phone, email or in-person to schedule an appointment and received an information letter in advance. The interviews were conducted in Dutch according to a semi-structured, pilot-tested topic list with open-ended questions on three related topics: valuable activities and life goals, goals of care in case of acute and/or severe disease in future life, and communication about these goals. This topic list was open to iterative revisions, which were made based on the results of our quantitative study [13] and on regular debriefing sessions with the research team in between the interviews (see Appendix 1 for a concise version of the final topic list). At the end of the interview, the researcher made in consultation with the participant an optional picture of something most valuable to the participant’s life, as pictures can be very powerful illustrations of what is important in life. In total, 42 interviews were carried out to collect sufficient data for analysis of each topic separately.

The interviews were conducted from July to November 2022. The interviews took place in-person in the participant’s (nursing) home or at another location of the participant’s choosing. If required or desired, participants were assisted by someone during the interview. Field notes were made during or immediately after the interview for recall of its context, and all interviews were audio-recorded, transcribed *ad verbatim* and anonymised. Given our frail study population, these transcripts were not returned to the participants for comments or corrections.

### Data analysis

We used thematic content analysis to identify and categorise recurrent themes in the data [19]. Two members of the research team independently coded interviews of five participants with varying frailty status and developed an initial coding tree on goals of care, which they judged for consistency of interpretation (V.v.d.K. and M.M.). Deductive and open, inductive coding were combined. In other words, we were interested in the meaning ascribed to goals of care of our quantitative study (*extending life*, *preserving quality of life*, *staying independent*, *relieving symptoms*, *supporting others*, *preventing hospital admission* and *preventing nursing home admission*) [13], as well as open to any other goals of care that emerged. Axial coding was applied thereafter to restructure the initial codes in overarching themes and four more interviews were double coded as a quality check. These preliminary themes were extensively discussed in the research team to ensure varied points of view and to reach consensus on their interpretation. To subsequently compare older people with varying frailty status, further analysis was done by V.v.d.K. in close collaboration with Y.D. This analysis phase focused on the severely frail participants first, based on the assumption they had most real-life experience with being acutely and/or severely ill, and then on comparing them to less frail participants. Interpretations were again extensively discussed within the research team until consensus was reached and no new themes emerged. Data saturation for this current qualitative analysis was reached after analysing 26 of the 42 interviews.

Atlas.ti version 23 was used to code and analyse the transcripts. SPSS Statistics version 25.0 (IBM Corp, Armonk, NY) was used for descriptive statistics on data from the quantitative questionnaire as appropriate. Quotations included in the paper were translated to English using forward translation.

### Patient and public involvement

Our Seniors Advisory Board (10 community-dwelling older people) was involved throughout the entire research cycle according to varying roles of the involvement matrix: listener, co-thinker, advisor, partner and decision-maker [20]. Their involvement especially improved the study’s inclusiveness for the heterogenous older population. Repeated discussion of themes also ensured incorporation of older people’s perspective in the data analysis. See Appendix 2 for more details on PPI [21].

## Results

The 26 participants had a median age of 82 years (ranging from 70 to 99). Fifty-eight percent were female, 58% had a lower or middle-level education and 65% lived alone. Seventeen (65%) participants lived independently at home and nine (35%) were in supportive care settings (two assistant living, seven nursing home). Regarding frailty status, 5 (19%) participants were classified as fit (self-reported CFS 1–3), 8 (31%) as mildly frail (self-reported CFS 4–5) and 13 (50%) as severely frail (self-reported CFS 6–8). The interviews had a median duration of 81 min (interquartile range 67 to 96). They took place in the participant’s (nursing) home (*n* = 23) and at a community centre, geriatric rehabilitation facility and hospital. Seven participants were assisted by someone. See Table 1 for more detailed descriptives per participant.

**Table 1:** Descriptives of the study population (*n* = 26).

Participant	Frailty status^a^	Age	Sex	Country of birth	Education^b^	Living alone	Residence	Experienced health problems^c^
P1	Fit	70	F	Indonesia	Middle	Alone	Independent	Mental, social
P2	Fit	72	F	Netherlands	Middle	Alone	Independent	
P3	Fit	78	M	Netherlands	Higher	Together	Independent	
P4	Fit	81	M	Curaçao	Higher	Together	Independent	Somatic
P5	Fit	84	F	Surinam	Lower	Alone	Independent	
P6	Mildly frail	71	M	Netherlands	Lower	Alone	Independent	Somatic, mental, social
P7	Mildly frail	74	M	Netherlands	Higher	Together	Independent	Somatic, mental
P8	Mildly frail	82	F	UK	Higher	Together	Independent	Somatic
P9	Mildly frail	82	F	Netherlands	Higher	Alone	Independent	Somatic, mental
P10	Mildly frail	82	M	Netherlands	Higher	Together	Independent	Somatic
P11	Mildly frail	83	F	Netherlands	Higher	Alone	Independent	Somatic, mental, social
P12	Mildly frail	84	F	Netherlands	Higher	Together	Independent	
P13	Mildly frail	88	M	Netherlands	Middle	Together	Assisted living	Somatic
P14	Severely frail	70	F	Netherlands	Lower	Alone	Nursing home	Somatic, functional
P15	Severely frail	75	M	Surinam	Lower	Alone	Nursing home	Somatic, mental, social, functional
P16	Severely frail	76	M	Netherlands	Higher	Together	Independent	Somatic, functional
P17	Severely frail	78	F	Netherlands	Lower	Together	Independent	Somatic, social, functional
P18	Severely frail	80	F	Surinam	Higher	Alone	Independent	Somatic, functional
P19	Severely frail	82	M	Netherlands	Lower	Alone	Nursing home	Somatic, functional
P20	Severely frail	83	F	Netherlands	Lower	Alone	Nursing home	Somatic, mental, social, functional
P21	Severely frail	86	F	Netherlands	Middle	Alone	Assisted living	Somatic, functional
P22	Severely frail	88	M	Netherlands	Lower	Alone	Independent	Somatic, mental, functional
P23	Severely frail	90	F	UK	Higher	Alone	Nursing home	Somatic, mental, social, functional
P24	Severely frail	90	M	Netherlands	Middle	Alone	Independent	Somatic, mental, social, functional
P25	Severely frail	93	F	Netherlands	Lower	Alone	Nursing home	Functional
P26	Severely frail	99	F	Netherlands	None	Alone	Nursing home	Somatic, mental, functional

Notes: ^a^Frailty status was defined according to a self-reported Clinical Frailty Scale (CFS): from fit (CFS 1–3) to mildly frail (CFS 4–5) and severely frail (CFS 6–8) [13]. ^b^Education was defined as the highest completed level of education according to the Dutch Verhage scale: lower = primary (vocational) education, middle = secondary (vocational) education and higher = higher vocational education or university [22]. ^c^Health problems were defined as experiencing ≥2 deficits during the past month out of the 4–7 deficits questioned per health domain of the Integrated Systematic Care for Older People questionnaire [23].

Three main themes were identified. The first two themes describe goals of care in case of acute and/or severe disease, which were independent of frailty status: (1) preserving well-being in one’s lifeworld through life goals and (2) additional goals of care related to the care. The third theme describes the way in which these goals of care were attained and adapted differed according to frailty status.

**Theme 1. Preserving well-being in one’s lifeworld through life goals** 

The participants’ lifeworld appeared to be a combination of their subjective, inner world and their relationships to the outside world, which comprised the life space and others. In case of acute and/or severe disease, the situation necessitated them to relate to the disease in question. A new facet in their lifeworld encompassing the illness and care context needed to be embedded on top of their previous disease experiences (Fig. 1.1).

**Figure 1:**
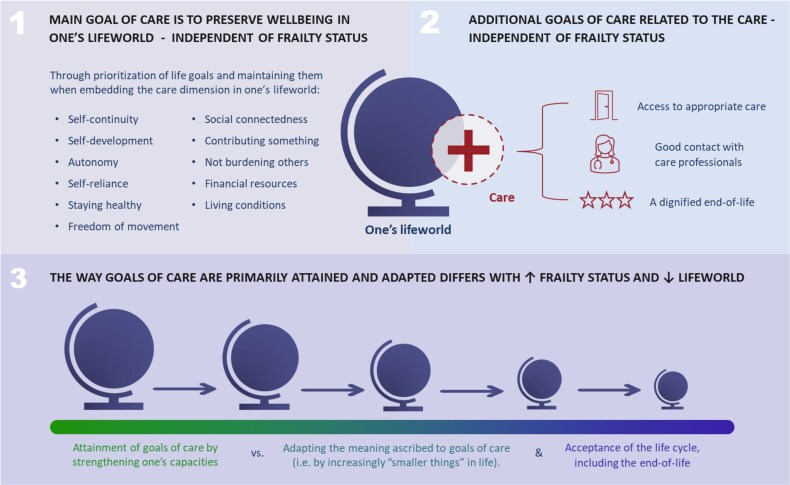
The role of frailty in older people’s perspectives on goals of care in case of acute and/or severe disease.

*‘You have bone marrow cancer, but we suspect more […] And then four days later, I was told: you also have lymph node cancer. Yes. So then your world is turned upside down, even though you are 82’.* (P9, mildly frail)

For all participants, the overarching goal of care in case of acute and/or severe disease was to preserve well-being in their lifeworld. In other words, to stay in a subjective state of quality and enjoyment of life despite disease. This sense of well-being in one’s lifeworld was determined by weights given to various life goals. In short, these life goals related to the self (self-continuity and self-development), to freedom (autonomy, self-reliance, staying healthy and freedom of movement) and to others (social connectedness, contributing something and not burdening others). Financial resources and living conditions were additional life goals that directly influenced well-being, as well as means to attain the other abovementioned life goals. See Table 2 for a more elaborate illustration per goal.

**Table 2:** Illustration of older people’s various life goals which determine well-being in one’s lifeworld.

Life goals	Description^a^	Quote
Self-continuity	To sense connection between one’s past, present and/or future self (e.g. upbringing, culture, religion, habits, etc.)	*‘They put a chair around the corner [of the apartment in the nursing home], because there’s a small patch of sunlight that shines here. And my mother is used to in her old house—she always sat in her spot with a sunbeam.’* (Caregiver of P23, severely frail)
Self-development	To achieve personal growth or experience new things (e.g. new knowledge or skills)	*‘When that task is completed [volunteer activity], I have a hunger for something new, and also a hunger for knowledge.’* (P12, mildly frail)
Autonomy	To have freedom to decide for oneself and to control one’s own actions	*‘Because I can do the things I want to do, you know. I don’t have any obligations. I don’t want that. I don’t want that anymore. I go swimming every Thursday. Nobody should interfere with my Thursday. No.’* (P18, severely frail)
Self-reliance	To depend on oneself and one’s own abilities to do things [e.g. in (instrumental) activities of daily living]	*‘That I can still do things and still ride a bike and go grocery shopping on the bike and go to a dance party and go crazy.’* (P1, fit)
Staying healthy	To feel as healthy as much as possible (e.g. bodily functions, by being fit, thinking clearly and relieving symptoms)	*‘I need to retain my cognitive capacities. […] If I can no longer recognize them [his family members], then I am a vegetable and I do not want to live as a vegetable.’* (P4, fit)
Freedom of movement	To have a certain degree of mobility and life space	*‘I would find it very distressing if I could no longer go outside, that would be the worst. I experience so much on the street. […] And I can also take the bus, which is right out front’* (P11, mildly frail)
Social connectedness	To feel connected to others by meaningful interactions, a sense of belonging or support	*‘I do the shopping […] You meet people, you get out. I find that very important. That you keep in touch with the outside world. […] you feel a bit dignified, that you still matter, that’s what it’s all about.’* (P17, severely frail)
Contributing something	To be meaningful or relevant to others or society (e.g. by being there or giving support)	*‘I have that neighbour downstairs, whom I read to. […] You can mean something to each other. Even if it’s just reading aloud for a bit or playing a game with someone.’* (P21, severely frail)
Not burdening others	To not trouble others with something that one regards unpleasant or difficult	*‘I have told my children: If I can’t do anything anymore, I won’t bother anyone, not even the children. I’ll stay somewhere in a care home. Whatever I get there, I’ll eat everything.’* (P5, fit)
Financial resources	To have financial resources to fund one’s needs and wishes	*‘And we also had a day with that wheelchair taxi, a day at the sea. I also find that so wonderful. […] it costs money and time, but it’s really nice.’* (P20, severely frail)
Living conditions	To live in a home and immediate surroundings with certain standards	*‘I was supposed to go to the fourteenth [floor], up there. But there are no living rooms there. And I did want a living room to sit together cosily.’* (P26, severely frail)

Notes: ^a^The descriptions clarify the common ground of quotes that were coded and categorised within a life goal. These categories emerged from the data and were sometimes in line with literature that was known to the researchers beforehand [13, 24].

Weights given to these life goals differed per person, but were independent of the participants’ frailty status. More specifically, we saw no differences in prioritisation of life goals by fit, mildly frail and severely frail participants, and subsequent well-being was evidently prioritised over life extension among all.

*‘I value quality of life. I don’t want to live no matter the cost.’* (P21, severely frail)

The participants wanted to maintain their life goals through the care that they received, as well as during receiving this care, which turned out to be essential for attaining person-centred care. One participant illustratively described this process of continuing his current life as maintaining a ‘steady state’:

*‘That you keep all kinds of things at a good level. That you manage to achieve and maintain a steady state.’* (P10, mildly frail)

Correspondingly, not being able to maintain personal life goals within the care impinged on experiencing person-centredness of care, as exemplified by a participant that valued autonomy:

*‘You actually have to be passive, and if you are assertive, then you are difficult. That is my experience with the medical side. […] And then I had the feeling: I am a patient, but I don’t matter.’* (P8, mildly frail)

Therefore, many participants eagerly engaged in advanced care planning and shared decision-making. In doing so, they had a broad perspective on care: not only considering medical care such as (invasive) treatment or palliative care, but e.g. also including informal, social, home and/or nursing care.

For some participants, however, current well-being was preserved by limiting engagement in their (future) care. For instance, by applying a wait-and-see attitude or by avoiding care.

*‘I think that it gives me joy in life by not constantly worrying.’* (P3, fit)

**Theme 2. Additional goals of care related to care** 

Upon emergence of care as facet in their lifeworld, three additional goals of care were mentioned by most severely frail, as well as less frail participants (Fig. 1.2): (2.1) access to appropriate care, (2.2) good contact with care professionals and (2.3) a dignified end-of-life to close one’s lifeworld. What participants considered ‘appropriate’, ‘good’ and ‘dignified’ in these goals of care differed per participant and was in line with the personal life goals they prioritised in their lifeworld.

2.1 Access to appropriate care

First, participants wanted to be able and allowed to access appropriate care. For some, such access was self-evidently within their control (e.g. by assertiveness or financial resources). However, many others felt that access to care was outside their circle of influence. Participants had trouble navigating the health and social care systems or considered current care options inappropriate to preserve their well-being. For instance, nursing homes predominantly offer one-bedroom apartments for individuals, while some participants wish to move with their partner and need two bedrooms. Participants also feared ageism in medical decision-making or that access to care would otherwise be denied or delayed (e.g. by long waiting lists).

*‘If I ask for an appointment [with the general practitioner]—Then the first question is not: When would you like it? But: Is it really necessary?’* (P10, mildly frail)

These concerns were rooted in negative care experiences and/or in media coverage on the rapidly ageing population, limited funding and staff shortages. Subsequent insecurities about getting access to appropriate care to preserve well-being in the future also negatively impacted current well-being.

*‘I am afraid… that the care for older people is going to decline […] 30 years ago, when there was an invention in the medical field, people would call hallelujah and it was to the benefit of the older generation, so they could live longer […] and now you occasionally hear statements that it is too expensive and it is becoming a problem.’* (P6, mildly frail)

2.2 Good contact with care professionals

Second, good contact with care professionals was an end in itself to preserve well-being, also in brief care relationships. For example, participants who valued autonomy and self-reliance described the need to be taken seriously, to be informed and to be an equal partner in decision-making.

*‘Because they [medical residents] are still learning, you can engage in more dialogue with them. […] That is, I think, the most important thing. That I can talk to a doctor, a healthcare provider, on an equal level.’* (P2, fit)

Participants who valued social connectedness mentioned the need to be understood, to feel kindness and compassion, and to build relationships.

*‘I think that I want to have a good relationship—I also make an effort for that, I do my best—and I get that in return, especially with the general practitioner.’* (P11, mildly frail)

Participants who did not want to burden others needed a proactive attitude of the professionals or preferred a reciprocal relationship in which they could contribute something (e.g. relieve workload).

*‘What I can [in terms of showering], I try to do myself, also to relieve them a bit, because we are all dealing with staff shortages. So, I think of it as a bit of a relief for them’* (P26, severely frail)

Furthermore, many participants expressed the profound need for undivided attention of professionals and to be seen as a person within a context instead of only as a patient with a disease.

*‘Someone who really takes the time. Not rush, rush, rush. Real time. And that doesn’t mean a lot of time. But concentrated time, attention.’* (Partner of P7, mildly frail)

2.3 A dignified end-of-life

Third, participants desired a dignified end-of-life in terms of dying and saying goodbye to one’s lifeworld. Regarding death, some participants specifically wished for a natural dying process (e.g. by symptom relieve or no resuscitation), while others wanted to intervene (e.g. by euthanasia or quitting any intake).

*‘Moreover, I have said: I only want to die once [no resuscitation].’* (P13, mildly frail)

Besides these medical procedures, many participants actively aimed to say goodbye to their lifeworld in a broader sense. For instance, participants deliberated how, where and with whom to spend their last time, as well as engaged in settling affairs, tidying their belongings and arranging their funerals in preparation for their end-of-life.

*‘Well, I, let’s say every few years, request a cost estimate from the undertaker, […] because it details exactly what they are paid for.’* (P4, fit)

Personal preferences in both the dying process and saying goodbye in this broader sense were among others aligned with self-continuity in (non)religious beliefs, the urge for autonomy and/or to not burden others (family and health care professionals).

*‘I try to start by tidying up a bit, because I have so much clutter, and I think, well, I can’t burden them with having to sort all of that out later.’* (P1, fit)

**Theme 3. Attainment and adaptation of goals of care differ according to frailty status** 

With a higher frailty status, the lifeworld of the participants eventually became smaller. It varied between participants which aspect(s) mostly diminished, the inner world and/or relationships to the outside world, and in what way these aspects diminished (i.e. in size and/or quality).

*‘I love my own square meters, I don’t go outside anymore, I don’t go anywhere anymore. […] I don’t mind. […] I’ve seen so much. I’ve seen the world.’* (P24, severely frail)

In any case, with a diminishing lifeworld, the way goals of care were predominantly attained and adapted differed (Fig. 1.3): (3.1) fit participants primarily strengthened their capacities to attain their goals of care and (3.2) participants with a higher frailty status primarily adapted the meaning ascribed to their goals of care and had higher acceptance of the life cycle.

3.1 Attainment of goals of care by strengthening capacities

Participants made personal efforts to stay in a good shape (e.g. physical or cognitive exercise), which strengthened the intrinsic capacity to maintain one’s goals. If desired or required, a range of tools and/or support from others was used to complement or substitute this capacity. Tools ranged from smartwatches and other digital devices to medical aids and adjustments in the living situation. Support from others covered the participants’ broad perspective on care (e.g. including domestic help or transport). What participants preferred to use out of these options to strengthen their capacities did not depend on their frailty status, but was again in line with the personal life goals they prioritised in their lifeworld.

*‘It neatly registers my current blood sugar levels [Freestyle Libre]. I thought that was wonderful. […] You give people control over their own lives, and that little device helps with that.’* (P3, fit)

Strengthening capacities in one way or another was the predominant strategy of fit older people to attain their goals of care. While participants with a higher frailty status could also attain their goals of care by strengthening capacities, adaptation was predominant.

3.2 Adaptation of goals of care through meaning and acceptance

First, the meaning ascribed to goals of care was adapted. In other words, the same life goals and subsequent goals of care could be attained by increasingly ‘smaller things’, as illustrated below:

*‘Being there, just being there. And beautiful cloudy skies, birds flying by, music that I occasionally hear. I can be very happy with that. And—that’s really it. But I find it enough. They are such small things and yet so significant.’* (P24, severely frail)

While many participants with a lower frailty status aimed to be self-reliant in all activities of daily living, a severely frail participant, for instance, attained her self-reliance by independently tidying her apartment and watering her plants. Similarly, autonomy was not necessarily attained by making all decisions oneself, but by deciding when one needs help with decision-making. See Table 3 for more illustrations of these ‘small things’ of frail participants. Fit participants could also enjoy such small things in their lifeworld, but the meaning they ascribed to their goals was overall more extensive.

**Table 3:** Illustration of relevance of the ‘small things’ with a higher frailty status and diminishing lifeworld.

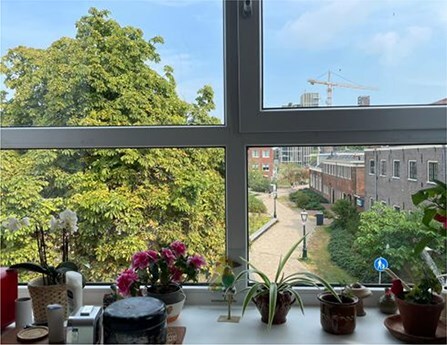	*‘Well, you can look outside nicely like this. I don’t know. So I enjoy it. […] This is nice, a nice view. What more could you want? […] “underneath the spreading chestnut tree” […] It’s a fantastic, beautiful view [at the nursing home].’* (P23, severely frail)
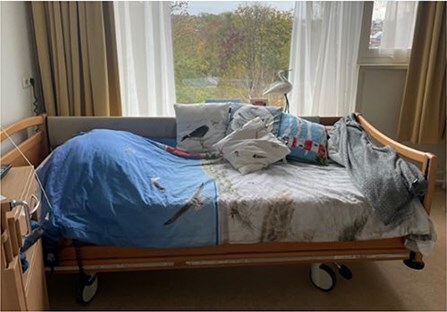	*‘We used to always go to the sea on vacation when the children were still little. It always kept pulling us […] it makes me happy. I’ve also decorated this room a bit with all sorts of things from the sea, you could say, even the sheet on my bed.’* (P20, severely frail)
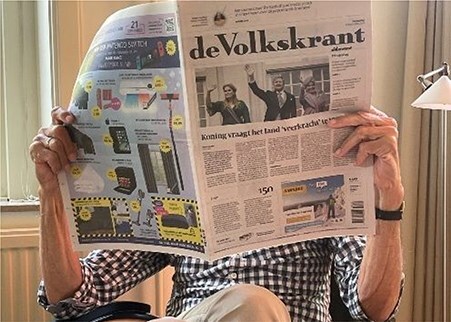	*‘The newspaper also provides some complicated pieces, longer pieces. And then I’m halfway through and I think: what is this actually about? [new symptom with early-stage dementia].’ […] Interviewer: ‘And what do you enjoy about reading the newspaper?’ Participant: ‘The football coverage or something, you know?’* (P7, mildly frail)
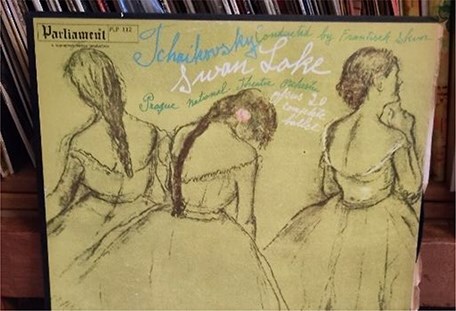	*‘I used to regularly go to concerts and to the theatre in Amsterdam [nowadays he is homebound]. […] On the computer, I spend a lot of time on YouTube, those music programs. I’m a music lover and especially of opera and classical music. And I find that such a privilege because I sit in the front row, I’m not bothered by people with popcorn stuff.’* (P24, severely frail)
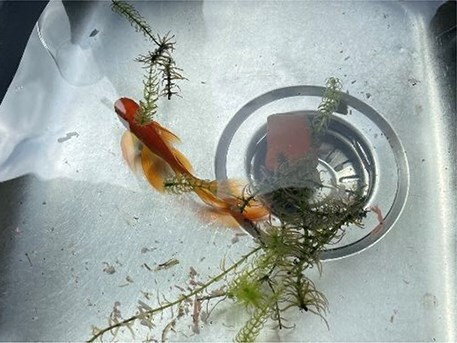	*‘I don’t leave here [home] because I need to be able to tinker around. […] I had just gotten out of bed and the little birds were without food again. Well, then I quickly go to feed them. And then the fish. […] If you don’t have that anymore, you’re worse off, don’t you think?’* (P22, severely frail)

Second, participants with a higher frailty status simultaneously adapted their goals of care by higher acceptance of the life cycle including disease, frailty and the approaching end-of-life. One did not have to, could not or should not wish for certain things anymore. For example, because disease symptoms were part of life, one’s purpose in life was fulfilled or resources could be better spent on one’s children.

*‘I sometimes say, my task is complete. The children can take over. Yes, we come and we go. We also leave, you know.’* (P20, severely frail)

Knowing that other older people were in a similar or sometimes even worse situation from one’s point of view also provided relief and promoted acceptance of the life cycle among participants.

*‘I have become so easygoing. I learned that from my sister. Even with wearing incontinence materials. I found it awful. But, well, what is it? We’re all human.’* (P21, severely frail)

## Discussion

In this qualitative study among the general older population, we found that their goals of care in case of acute and/or severe disease was not related to frailty status. Fit, mildly frail and severely frail older people wanted to (theme 1) preserve well-being in their lifeworld and (theme 2) wanted to have access to appropriate care, good contact with care professionals and a dignified end-of-life. Maintaining personal life goals through and during care was essential for these goals of care. In this context, older people extensively considered care to be much broader than medical care. By contrast (theme 3), the way older people attained and adapted their goals of care was related to frailty status. Older people with a higher frailty status ascribed a different meaning to the same goals of care and had higher acceptance of the lifecycle compared to fit older people, who primarily strengthened their capacities to attain goals of care.

The first two themes on which goals of care matter to older people with varying frailty status extend our preceding quantitative study by showing the meaning and interrelationships of their shared goals of care [13]. For instance, preserving well-being in one’s lifeworld or quality of life is not only very important, but is the overarching goal of care from the perspective of the older people themselves. Access to appropriate care, good contact with care professionals and a dignified end-of-life were not incorporated in measurement tools of previous studies relating goals of care to frailty status [13–16]. The importance of how one is approached by professionals did emerge previously in studies on what mattered most to patients regarding care in the emergency department [25, 26] and aspects like preparing for death and completing unfinished business emerged in studies regarding the end-of-life [27]. To the best of our knowledge, there is no previous literature on access to care already being a goal of care to older people, which may therefore be rooted in recent events such as the COVID-19 pandemic [28]. In any case, our findings suggest generalisability of these goals of care across frailty levels and care settings.

Life goals were shown to be the main drivers of older people’s goals of care. The life goals in our study were related to the self, to freedom and to others besides instrumental goals like financial freedom and living conditions. These life goals are in line with existing literature on aspects of quality of life in old age [29, 30], as well as on connectedness [24], successful ageing [31] and broad operationalisations of health [32]. The profound wish of older people in general to maintain their life goals in case of acute and/or severe disease is explicable when considering the substantial amount of time older people spend on getting care [33].

Prioritisation of these life goals was personal and independent of frailty status, in contrast to earlier findings of Puts *et al*. [30]. Their interviews showed that fit older people mainly prioritised health, while frail older people prioritised social contacts as the most important factor for quality of life in response to poorer health. From our participant’s perspectives, however, the personal prioritisation of life goals were independent of frailty status. This could mean that personal prioritisation of life goals is stable over time and across circumstances, which may reflect one’s outlook on life or disposition [24, 34]. Stability of personal life goals and subsequent goals of care despite higher frailty status is in line with the well-known ‘disability paradox’ [29, 31, 35], which describes that general well-being or life satisfaction remains stable despite increasing limitations in functioning.

In the third theme, we showed that the way older people predominantly attained and adapted their goals of care did differ with higher frailty status, which may be explained by experiencing more health-related barriers to goals of care [36]. The ways primarily applied by our frail participants (theme 3.2) were also touched upon by Etkind *et al*., who found that if ‘getting back to normal’ was not possible following acute illness, frail older people sought ‘a new normal’ (e.g. by reconceptualisation of normality or acceptance) [37]. We specified that this reconceptualisation meant to our participants that their life goals and subsequent goals of care were attained by increasingly ‘smaller things’ in life, which is called ‘scale recalibration’ [35]. This adaptation enabled older people with higher frailty status to maintain the same type of goals and is in line with ‘response shift theory’ [37, 38], which describes how objective changes in health do not necessarily result in expected changes in self-perceived outcomes. It may therefore explain the difference between the perspectives of older people themselves and professionals on goals of care in relation to frailty status, as mentioned in the introduction. The acceptance may also explain the slightly less importance frail older people attached to individual goals of care in previous questionnaire-based studies compared to fit older people [13, 16]. In addition, we add to the existing literature by showing the ways primarily applied by fit older people (theme 3.1), who were more focused on assimilative coping (adjusting circumstances to expectations, e.g. by health promotion) instead of accommodative coping (adjusting expectations to circumstances, as described above) [29].

Taken together, our findings show the possibilities of diving into the perspective of older people to improve person-centredness of care. Older people with varying frailty status could be treated similarly in goal-setting, as their goals of care are more homogenous and their prioritised life goals are more personal than based on frailty status. In complex decision-making, life goals rather than frailty could therefore be the most important factor in goals of care for older patients. Assessing frailty, however, is still an important aspect in complex decision-making with older patients because of the well-investigated value in predicting outcomes like functional decline, quality of life and mortality and advance care planning. So, in complex decision-making, assessment of both frailty and personal goals of care is needed. Eliciting the personal meaning ascribed to life goals could serve as a data-based starting point in SDM, independent of frailty status [6]. Also, as older people want to continue their current life in line with these life goals rather than life extension *per se*, expected outcomes of medical treatment need to serve life goals and death of an older patient may not necessarily be a failure [17]. This means that providing ‘the best’ medical treatment may even be a missed opportunity to provide the best person-centred care for fit and frail older patients, especially when acknowledging older people’s broad perspective on care. As the way goals of care were attained and adapted did differ between fit and frail older people, different attention of a wide range of care professionals might be needed to support their predominant attainment or adaptation strategy. Educating care professionals in concepts like the disability paradox and response shift may therefore be desirable. In addition, care professionals ought to be aware that having a personalised care experience is a goal of care in itself to older people besides the outcomes of the care they receive, also in brief relationships, and policy-makers need to facilitate access to care (e.g. by financial compensation for goal-setting in SDM).

Embedding the current study in a mixed-methods design provided multiple strengths [13]. First, this enabled us to explore the meaning and interpretation of quantitative results in this, to our knowledge, first qualitative study on the relationship between older people’s goals of care and frailty status. Second, the in-depth interviews gave a voice to older people themselves to describe new domains in their words. Third, purposive sampling from the broad community sample of our quantitative study enabled inclusion of a varied population, especially regarding frailty in old age. In addition, all of these aspects were strengthened by the ongoing close collaboration with our Seniors Advisory Board.

On the other hand, it could be seen as a limitation that our results regard hypothetical decision-making. In case of actual disease in future life, older people might act differently than expressed during the interviews. However, we consider it unlikely that this design affected our results related to frailty status, as both our fit and frail participants spoke from extensive disease experience (either personal and/or from others), and our results were in line with more clinical studies [14–16, 37]. Next to this, the generalisability may differ internationally by different cultures. We used a self-reported CFS, which should be studied further for construct validity. Since frailty prevalence was similar to self-reported frailty in a large population study in the Netherlands and showed the same trends in sociodemographics, we consider it unlikely that the use of the self-reported CFS affected our results [13]. Lastly, the transferability of our findings to, for instance, older people with severe cognitive impairment or diverse older migrants would be worthwhile future research.

In conclusion, goals of care that older people want to attain in case of acute and/or severe disease are independent of frailty and driven by life goals. Older people with varying frailty status could therefore be treated similarly in goal-setting, namely person-centred, and life goals rather than frailty could be the most important factor in complex care decisions. As fit and frail older people do differ in the way they attain and adapt their goals of care, different support may be needed in this respect.

## Supplementary Material

aa-24-1721-File002_afaf022

## Data Availability

The data are not publicly available due to privacy concerns. The data that support the findings of this study are available upon reasonable request from the corresponding author.
