# Supplementary material for: A qualitative study regarding older people’s goals of care in relation to frailty status: finding meaning in ‘smaller things’ in life
Source: Age Ageing. 2025 Feb 20;54(2):afaf022. doi: 10.1093/ageing/afaf022 (PMC11840562; doi:10.1093/ageing/afaf022)
Supplement: aa-24-1721-File002_afaf022 [file aa-24-1721-file002_afaf022.docx]

**A qualitative study regarding older people’s goals of care in relation to frailty status:**

**finding meaning in “smaller things” in life - Appendices**

van der Klei VMGTH, van den Bos F, Mooijaart SP, Julien AG, Maissan MJE, van Raaij BFM, Festen J, Gussekloo J, Drewes YM, on behalf of the COVID-19 Outcomes in Older People (COOP)-consortium

**Content list**

Appendix 1: Concise version of the topic list of the COOP-study on goals of care (forward-translation).

Appendix 2: Patient and Public Involvement (PPI) in the COOP qualitative substudy on older people’s goals of care according to the GRIPP2 short form [1].

| **Appendix 1.** Concise version of the topic list of the COOP-study on goals of care (forward-translation). |
| --- |
| **Topic 1. Valuable activities and life goals**   - Could you describe which activities are currently valuable to you in daily life? - Why are these activities important or what do you attain with these activities? - How did these activities evolve over time? If applicable, after [disease or life event]? - If applicable, what did you need to adapt your valuable activities in the past? - What matters regarding your life in the future? Which goals, wishes or desires do you have? - Out of your valuable activities, what do you preferably want to preserve in the future? - Out of your valuable activities, what do you minimally need for a good quality of life (QoL)? |
| **Topic 2. Goals of care in case of acute and/or severe disease**   - What is your experience with being ill? If applicable, with acute and/or severe disease? - What were important outcomes to you after [your disease and care experience]? - When being ill, “goals of care” are often discussed. How do you define goals of care? - What if you would (again) become acutely or severely ill, what would matter to you regarding your medical care and treatment? Which goals would you have regarding your care and treatment? Or what outcomes should minimally be attained with your care and treatment? - What are you willing to give up in case of care or treatment to preserve your QoL? - “Independence” is often discussed in health care. How do you define (in)dependence? - If applicable, how did you end up living in a nursing home? How did you regard this in the past? |
| **Topic 3. Preferred communication about these goals**   - What is your experience with sharing your goals of care with others? If applicable, with your family, other medical representatives and with (un)known health care professionals? What facilitated or hampered you in sharing these goals? If applicable, what role did others have? - How do you prefer sharing your goals of care in case of future acute and/or severe disease? What if you cannot share your goals yourself (e.g. delirium)? What do you need from others? - In what way are you now considering the chance of becoming acutely and/or severely ill? To what extent did the COVID-19 pandemic influence this awareness? How do you prepare or how should health care professionals be best prepared to care for you in case of a new pandemic? |

| **Appendix 2.** Patient and Public Involvement (PPI) in the COOP qualitative substudy on older people’s goals of care according to the GRIPP2 short form [1]. | |
| --- | --- |
| **1. Aim**  Report the aim of PPI in the study | To give voice to older people who are ‘experts by experience’ based on their age, diverse medical histories and experience with the COVID-19 pandemic in the Dutch context by collaborating with them in all research stages of this exploration of goals of care in relationship to frailty status. |
| **2. Methods**  Provide a clear description of the methods used for PPI in the study | Ten older people were recruited to participate in the COOP Seniors Advisory Board: median age 75 years old (IQR 70-78), 70% female and 100% higher educated*. They had diverse medical experiences (e.g. history of COVID-19 or other disease and ranging from fit to mildly frail) and professional backgrounds (health care and research, anthropology, education and finances). 70% also represented another senior organization spread across the Netherlands.  Throughout the project the Board participated according to all five roles of the Involvement-Matrix: listener, co-thinker, advisor, partner and decision-maker [2]. Their chairman was part of the steering committee of the larger COOP-consortium and was co-leader of this substudy on goals of care. He was involved from the very beginning in defining the research question and drafting the grant application. Subsequently, the Board was involved in the study design, data collection and interpretation of the results of both the preceding quantitative substudy [3], as well as this qualitative substudy. They will also be involved in dissemination of a lay summary of our mixed-methods findings via senior organizations.  The Board had bi-monthly meetings either online or in person with at least one junior and one senior researcher from this substudy involved. |
| **3. Results**  Outcomes—Report the results of PPI in the study, including both  positive and negative outcomes | The COOP Seniors Advisory Board influenced this substudy as follows:   - The Board improved the heterogeneity of our sampling frame by revising and distributing the preceding quantitative questionnaire. - The Board hypothesized that the Clinical Frailty Scale (CFS) primarily reflected somatic health instead of a more holistic concept of frailty, while they regard psychosocial well-being as very important when indicating heterogeneity in old age as well. To complement our primary exposure (frailty), diversity in mental and social health problems was therefore also considered during purposive sampling. - The Board participated in pilot interviews to train the interviewers and to experience the initial topic list from the lay perspective, after which minor revisions were made to improve its comprehension. - The Board was repeatedly involved during the data analysis in discussions about the (preliminary) themes to complement the professional perspective of the research team. They particularly recognized and stressed the utmost importance of well-being (theme 1), the additional goals of care from the older people’s perspective that inductively emerged from the data (theme 2), and the shift in meaning ascribed to goals of care with higher frailty status / diminishing lifeworld (theme 3). - Their interpretation of results was incorporated in the discussion based on above-mentioned meetings and co-authorship of the chairman. - The Board proposed additional strategies to disseminate our mixed-methods results and to implement them in medical practice, which will be included in a subsequent implementation grant proposal.   For pragmatic reasons, the Board was not directly involved in conducting the interviews with participants and in reading or coding the transcripts. |
| **4. Discussion and**  **Conclusions**  Outcomes—Comment on the extent to which PPI influenced the study  overall. Describe positive and negative effects | PPI involvement of older people in this substudy encompassed varying roles of the Involvement-Matrix throughout all research stages and especially enhanced the study’s inclusiveness for the heterogenous older population. Furthermore, the qualitative nature of this substudy allowed for repeated involvement of the Board in the discussion of evolving themes, which ensured the incorporation of older people’s perspective during the data analysis and thereby, improved the relevance of our results. |
| **5. Reflections,**  **critical perspective**  Comment critically on the study, reflecting on the things that went well  and those that did not, so others can learn from this experience | The involvement of the Seniors Advisory Board was overall very positive. The Board was rapidly established via the large network of the COOP-consortium. The extensive previous experience of the chairman and senior researchers with PPI, together with professional training in PPI for the junior researcher involved, facilitated fruitful collaboration throughout the project. Digital skills of the Board facilitated convenient online meetings, especially relevant during the COVID-19 pandemic, and any expenses could be reimbursed (e.g. for in-person meetings).  Severely frail older people were unfortunately not directly included in the PPI. Therefore, the Board might have been less equipped to communicate their perspectives. Additionally, as the Board also participated in the larger COOP-consortium, their commitment was relatively time-consuming. |
| **Notes**: ^*^According to the Dutch Verhage Scale on educational attainment.  **Abbreviations**: COOP, COVID-19 Outcomes in Older People consortium; GRIPP2, Guidance for Reporting Involvement of Patients and the Public version 2; IQR, interquartile range. | |

**REFERENCES**

1. Staniszewska S, Brett J, Simera I, et al.; GRIPP2 reporting checklists: tools to improve reporting of patient and public involvement in research. *BMJ* 2017;**358**:j3453. doi: 10.1136/bmj.j3453.

2. Smits DW, van Meeteren K, Klem M, et al.; Designing a tool to support patient and public involvement in research projects: the Involvement Matrix. *Res Involv Engagem* 2020;**6**:30. doi: 10.1186/s40900-020-00188-4.

3. van der Klei VMGTH, Drewes YM, van Raaij BFM, et al.; Older people’s goals of care in relation to frailty status—the COOP-study. *Age and Ageing* 2024;**53**(5). doi: 10.1093/ageing/afae097.
